# Plasma-activated medium promotes autophagic cell death along with alteration of the mTOR pathway

**DOI:** 10.1038/s41598-020-58667-3

**Published:** 2020-01-31

**Authors:** Nobuhisa Yoshikawa, Wenting Liu, Kae Nakamura, Kosuke Yoshida, Yoshiki Ikeda, Hiromasa Tanaka, Masaaki Mizuno, Shinya Toyokuni, Masaru Hori, Fumitaka Kikkawa, Hiroaki Kajiyama

**Affiliations:** 1 Department of Obstetrics and Gynecology, Nagoya University Graduate School of Medicine, Nagoya, Japan; 2 Bell Research Center for Reproductive Health and Cancer, Nagoya University Graduate School of Medicine, Nagoya, Japan; 3 Center for Low-temperature Plasma Sciences, Nagoya University, Nagoya, Japan; 4 Center for Advanced Medicine and Clinical Research, Nagoya University Hospital, Nagoya, Japan; 5 Department of Pathology and Biological Responses, Nagoya University Graduate School of Medicine, Nagoya, Japan

**Keywords:** Endometrial cancer, Biomedical engineering

## Abstract

The biological function of non-thermal atmospheric pressure plasma has been widely accepted in several types of cancer. We previously developed plasma-activated medium (PAM) for clinical use, and demonstrated that PAM exhibits a metastasis-inhibitory effect on ovarian cancer through reduced MMP-9 secretion. However, the anti-tumor effects of PAM on endometrial cancer remain unknown. In this study, we investigated the inhibitory effect of PAM on endometrial cancer cell viability *in vitro*. Our results demonstrated that AMEC and HEC50 cell viabilities were reduced by PAM at a certain PAM ratio, and PAM treatment effectively increased autophagic cell death in a concentration dependent manner. In addition, we evaluated the molecular mechanism of PAM activity and found that the mTOR pathway was inactivated by PAM. Moreover, our results demonstrated that the autophagy inhibitor MHY1485 partially inhibited the autophagic cell death induced by PAM treatment. These findings indicate that PAM decreases the viability of endometrial cancer cells along with alteration of the mTOR pathway, which is critical for cancer cell viability. Collectively, our data suggest that PAM inhibits cell viability while inducing autophagic cell death in endometrial cancer cells, representing a potential novel treatment for endometrial cancer.

## Introduction

Endometrial cancer is the most common gynecologic malignancy in Japan, with an estimated 13,600 new cases diagnosed in 2015^1^. The incidence of new cases is believed to have increased over the last few decades due to increased levels of obesity, hypertension, and diabetes mellitus, particularly in South Africa and several Asian countries including Japan^1,2^. Although the majority of endometrial cancer patients are diagnosed in the early stage and outcomes are generally favorable because of the typical symptom of irregular vaginal bleeding in women of menopausal age, the prognoses of patients in advanced stages not amenable to localized therapies remain poor^1^. Indeed, a frequent pattern of progression and recurrence is peritoneal metastasis, which is often resistant to systemic chemotherapy^3^. In addition, cytotoxic agents for endometrial cancer are relatively limited compared with other gynecologic malignancies. Thus, there is an increasing demand for novel approaches to peritoneal dissemination.

For the past decade, nonequilibrium atmospheric pressure plasma (NEAPP) has been studied for its clinical applications such as sterilization, wound healing, and cancer therapy^4–6^. NEAPP represents a partially ionized gas with energy mainly accumulated as free electrons^7^. Recent studies have revealed that in addition to the direct irradiation of NEAPP to bacteria or tissues, indirect plasma treatment with plasma-activated medium (PAM) and plasma-activated lactate (PAL), which are activated by NEAPP, exhibit anti-tumor activity in several types of cancer, including ovarian, pancreatic, colorectal and gastric cancers^4,8–13^. The molecular mechanism of the anti-tumor effects triggered by PAM have been investigated, and the intracellular accumulation of reactive oxygen species (ROS) induced by PAM is presumed to play an important role^9,10^. In addition, our colleagues reported that the intraperitoneal administration of PAM effectively inhibits the peritoneal dissemination of gastric cancer and ovarian cancer in a mouse model, which may be applicable in clinical situations^14^. Despite extensive studies regarding NEAPP and PAM, their anti-tumor effects against endometrial cancer have not been investigated.

Autophagy, a process by which cells degrade their own cytoplasmic components and organelles, is also known as programmed cell death different from apoptosis^15^. Abnormalities of autophagy are closely related not only to neurodegenerative diseases and metabolic disorders, but also to carcinogenesis in various types of cancer^15,16^. Recent studies on various substances have reported that autophagy plays a paradoxical role in anti-tumor therapy. Induction of autophagic cell death by small molecules contributes to the enhancement of anti-tumor activity of chemotherapy or radiation therapy, whereas autophagy-dependent anti-apoptotic responses induced by chemotherapy have negative impacts on anti-tumor treatment through inhibition of the mTOR pathway^17,18^. To our best knowledge, no association between anti-tumor effects of PAM and autophagy has been reported.

The aim of this study was to evaluate the antitumorigenic effects of PAM on endometrial cancer cells and to elucidate the underlying mechanism. To this end, the suppressive effects of PAM on endometrial cancer cell viability *in vitro* were examined and autophagy was a part of the suppressive effects of PAM.

## Results

### PAM was shown to inhibit the cell viability of endometrial cancer depending on the cell type and dilution ratio

**Figure 1:**
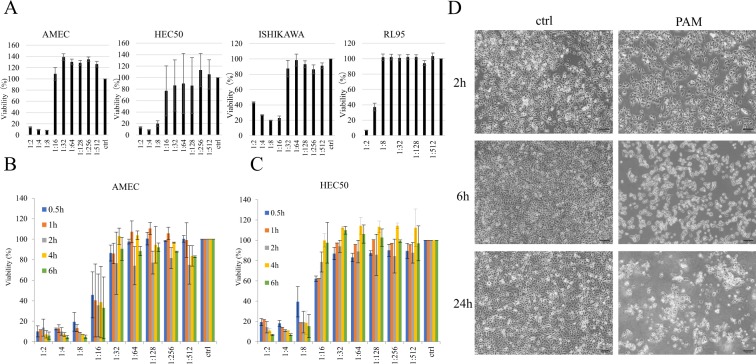
Plasma-activated medium (PAM) inhibits the viability of endometrial cancer cells, depending on the cell type, PAM dilution ration, and duration time of PAM treatment. (**A**) The sensitivities of AMEC, HEC50, ISHIKAWA, and RL95 cells to PAM were evaluated by Cell Viability Assay. (**B**) Cell viability using Cell Viability Assay at different PAM concentration and duration time of PAM treatment in AMEC cells. (**C**) Cell viability using Cell Viability Assay at different PAM concentration and duration time of PAM treatment in HEC50 cells. (**D**) Morphological changes in AMEC cells at 2 h, 6 h, and 24 h after 1:4 PAM treatment. Data from Cell Viability Assay are presented as mean ± SD. Three replicates were performed.

To evaluate the anti-proliferative effects of PAM on endometrial cancer cells, we first performed cell viability assays in a total of four endometrial cancer cell lines: AMEC, HEC50, ISHIKAWA, and RL95. Figure 1A shows the viability of the cells treated with gradient ratios of PAM for 24 h. PAM treatment decreased the percentage of viable cells in all endometrial cancer cell lines in a concentration-dependent manner. AMEC and HEC50 cells demonstrated a higher sensitivity to PAM than the other cell lines. Therefore, we decided to use these cell lines for subsequent experiments. As shown in Fig. 1B,C, 0.5 h treatment with PAM resulted in a considerable decrease in cell viability for both AMEC and HEC50 cell lines. Morphological changes in AMEC cells were induced by PAM within 2–24 h and were similar to the morphology often observed in cell death (Fig. 1D). Collectively, our results indicated that PAM had the potential to suppress cell viability and induce cell death in endometrial cancer cells.

### PAM induces cell death in a time-dependent manner in endometrial cancer cells

**Figure 2:**
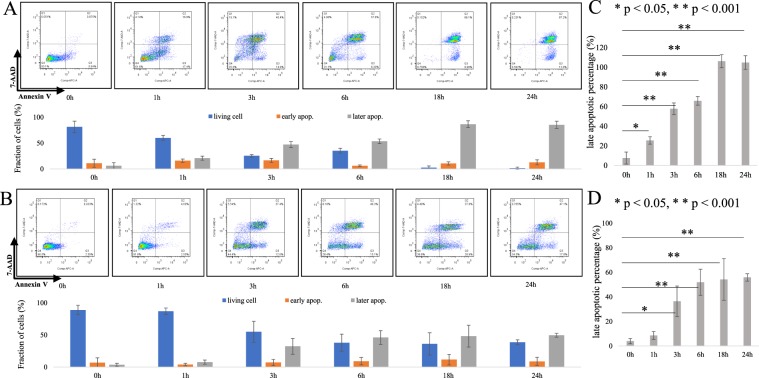
Plasma-activated medium (PAM) induces cell death in a time-dependent manner in endometrial cancer cells. (**A**) The fractions of living cells, early apoptotic cells (early apop) and late apoptotic cells (late apop) in AMEC cells were determined by Annexin V/7-AAD staining assays. (**B**) The fractions of living cells, early apoptotic cells (early apop) and late apoptotic cells (late apop) in HEC50 cells by Annexin V/7-AAD staining assays. (**C,D**) The status of AMEC (**C**) and HEC50 (**D**) were quantified from triplicate samples. All data from Annexin V/7-AAD staining assays are presented as mean ± SD. Three replicates were performed.

We next performed Annexin V/7-AAD staining assays to evaluate whether PAM effectively induced cell death in endometrial cancer cells. Treatment with PAM increased the fraction of Annexin V positive cells in both AMEC and HEC50 cells (Fig. 2A,B). In AMEC cells, early apoptotic cells increased from 10.9% of control to 12.7% with 24 h PAM treatment; however, this difference was not significant (Fig. 2A). On the other hand, late apoptotic cells were significantly increased from 6.1% of control to 85.3% with 24 h PAM treatment (*p* < 0.05) (Fig. 2C). In HEC50 cells, although early apoptosis was not significantly induced (from 6.8% of control to 8.7% with PAM treatment), late apoptosis was significantly increased from 3.5% of control to 49.6% by PAM treatment (*p* < 0.05) (Fig. 2D). These results indicated that PAM effectively induces cell death in a time-dependent manner in endometrial cancer cells.

### PAM modulates cell cycle progression and intracellular ROS accumulation of endometrial cancer cells

To further investigate unknown tumor-suppressive mechanisms of PAM on endometrial cells, cell cycle analysis was performed. Cell cycle distribution of AMEC and HEC50 cells were examined 24 h after PAM treatment by flow cytometry in different PAM dilution ratio. As shown in Supplementary Fig. 1, cells exposed to PAM exhibited G2/M cell cycle accumulation. Statistical significances were observed the G2/M-phase arrest in all the PAM concentrations. Next, As it was reported that PAM induces intracellular ROS accumulation, we investigated intracellular ROS accumulation using 5-6-chloromethyl-2′7′-dichlorodihydroflorescein diacetate, acetyl ester (CM-H_2_DCFDA). PAM treatment for 24 h promoted intracellular ROS accumulation in AMEC cells (Supplementary Fig. 2). These results demonstrated that PAM modulates cell cycle progression and intracellular ROS accumulation of endometrial cancer cells.

### PAM induces autophagy of endometrial cancer cells

**Figure 3:**
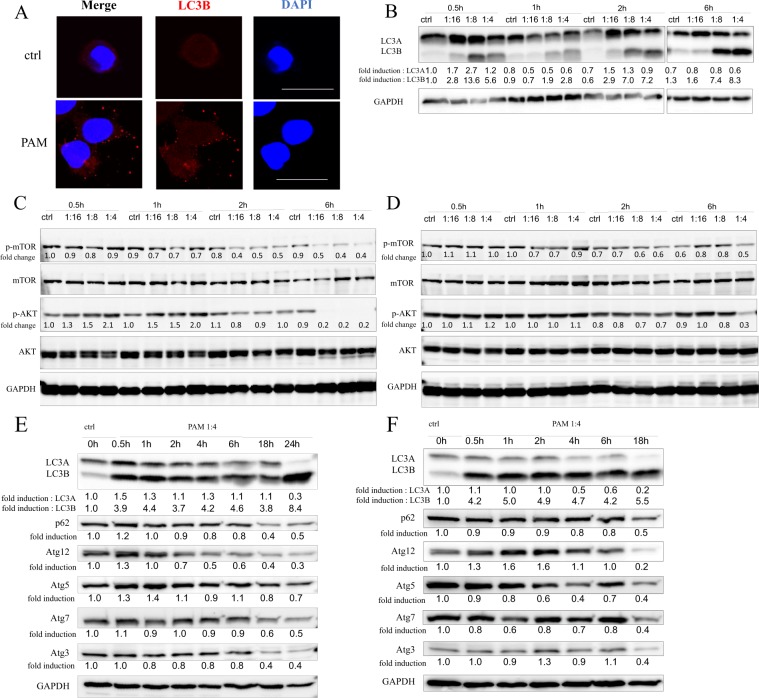
Plasma-activated medium (PAM) induces autophagy of endometrial cancer cells. (**A**) Representative images of Immunocytochemistry staining by LC3B with or without PAM treatment for AMEC cells. (**B**) Expression levels of LC3 protein after 0.5–6 h PAM treatment in AMEC cells were evaluated by Western blotting in different concentrations. (**C**,**D**) Effect of PAM on mTOR and AKT activation after 0.5–6 h in different PAM concentrations in AMEC (**C**) and HEC50 (**D**) cells. (**E**,**F**) Expression levels of LC3, p62 and ATG family proteins after 1:4 PAM treatment in AMEC (**E**) and HEC50 (**F**) cells at 0–24 h. The numbers indicated the density value normalized to GAPDH using the ImageJ software. The density value of phosphorylated form was expressed as the ratio to the density value of total protein. The original blots are presented in Supplementary Fig. 3.

To elucidate the mechanism of cell death induced by PAM, autophagosomes were probed by staining for the LC3B protein using cell immunofluorescence. Immunocytochemistry staining revealed that PAM treatment increased intracellular autophagosomes in AMEC cells (Fig. 3A). To detect the expression levels of autophagy-related proteins, Western blotting was performed. As shown in Fig. 3B, protein levels of LC3B increased with different durations of PAM treatment in a concentration-dependent manner in AMEC cells. Furthermore, in AMEC cells, PAM led to a decrease in phosphorylated mTOR and phosphorylated AKT protein levels after 2–6 h treatment (Fig. 3C). In HEC50 cells, phosphorylation of mTOR and AKT slightly decreased 1–6 h after administration of 4-fold diluted PAM (Fig. 3D). In addition to 24 h treatment, increased LC3B expression was found for 1–18 h PAM treatment in both AMEC and HEC50 cells (Fig. 3E,F). On the other hand, the expression of p62 and ATG family proteins was attenuated depending on the duration of PAM treatment in AMEC and HEC50 cells. These results showed that PAM induces the activation of LC3B and the decline of autophagy-related proteins, and phosphorylation of mTOR and AKT is inhibited by PAM.

### The autophagy inhibitor, MHY1485, rescues autophagy and cell death induced by PAM

**Figure 4:**
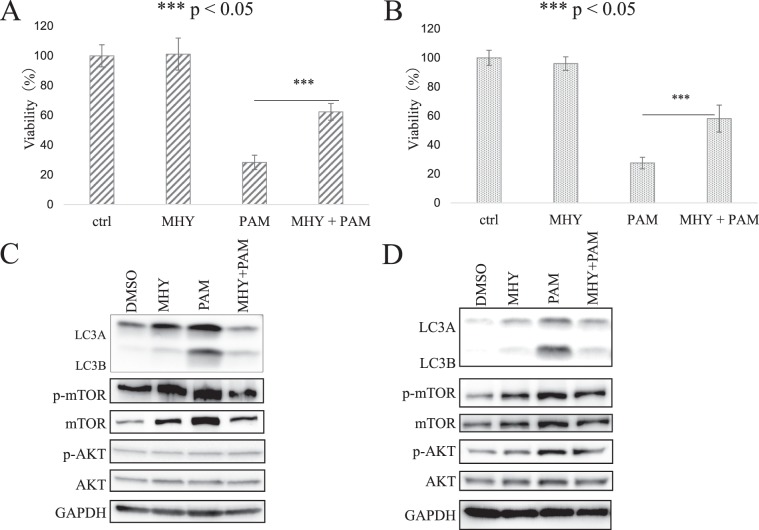
Autophagy inhibitor, MHY1485 (MHY), inhibits biological function of plasma-activated medium (PAM). (**A,B**) Reduction of cell viability by PAM treatment was rescued by MHY1485 in AMEC (**A**) and HEC50 (**B**) cells. (**C**,**D**) Attenuation of PAM effect on expression of LC3, but not mTOR, and AKT activation by MHY1485 in AMEC (**C**) and HEC50 (**D**) cells. The density value of each band was normalized to GAPDH by the ImageJ software. Phosphorylated form was expressed as the ratio of density value of phosphorylated form to the density value of corresponding total protein level. The original blots are presented in Supplementary Fig. 3.

To determine whether autophagy induced by PAM treatment actually induces decreased cell viability, we used the autophagy inhibitor MHY1485. After pretreatment with MHY1485, the reduced cell viability by PAM treatment was significantly rescued in both AMEC and HEC50 cells (Fig. 4A,B). Furthermore, Western blot analysis revealed that pretreatment with MHY1485 decreased the expression of LC3B that was increased by PAM treatment (Fig. 4C,D). On the other hand, although increased expression levels of mTOR and AKT by PAM treatment was attenuated by pretreatment with MHY1485, no objective differences were found in phosphorylation of mTOR and AKT. Our results suggested that the anti-tumor effects of PAM are partly mediated by autophagy activation.

## Discussion

NEAPP is widely considered as a next-generation cancer therapy, and the underlying mechanism of NEAPP has been widely investigated. However, the clinical application of NEAPP to cancer is still at the basic research stage. Direct exposure of NEAPP has the limitation that it only reaches the very surface of the tumor in a distance-dependent manner. Therefore, as a method that can extensively reach cancer cells in the peritoneal cavity, we have devised PAM and conducted basic research to utilize PAM for clinical use as an indirect plasma therapy. Interestingly, our colleagues and we have demonstrated the anti-tumor effects of PAM against several types of cancer using an *in vivo* model of peritoneal metastasis^8,11,19^. These findings suggest that PAM may be a novel option for the treatment of peritoneal metastasis. In this study, we confirmed the anti-tumor effects of PAM on endometrial cancer. Furthermore, previous studies regarding the direct exposure of NEAPP have demonstrated that the anti-tumor effects are due to mechanisms such as the induction of apoptosis, the inhibition of migration and invasion, and the promotion of cell cycle arrest^20,21^. Although we previously reported that PAM effectively inhibits ovarian cancer plantation in human peritoneal mesothelial cells, the mechanism of the anti-tumor effects of PAM is unclear compared with that of the direct exposure of NEAPP^8^. However, our current results indicated the possibility that autophagy may be a novel mechanism of PAM.

In the current study, we demonstrated that only a short time exposure of 0.5 h could exhibit sufficient anti-tumor effects *in vitro* on endometrial cancer cells. Previous reports revealed that longer treatments with direct exposure of NEAPP or PAM resulted in significantly lower viability in cancer cells^8,22^. In addition, Takeda *et al*. revealed that not only adherent cells, but also floating cells, presented a decrease in adhesion capacity^19^. When applying PAM as part of a clinical treatment to prevent peritoneal metastasis, a shorter exposure time with PAM to the intraperitoneal cavity is necessary to minimize side effects on normal cells. Our results indicate that upgraded devices can produce stronger PAM than previous devices, thus stronger PAM is much more applicable for clinical use from the perspective of its anti-tumor effects.

The induction effect of PAM on endometrial cancer cell death was confirmed in our study. Xiang L *et al*. reported that PAM could selectively reduce the cell viability of triple negative breast cancer cells^23^. Cell death was also induced by treatment with PAM in other types of cancers, including pancreatic cancer, glioblastoma, squamous head and neck cancer, and melanoma^24–26^. These authors presumed that the underlying mechanism of cell death was related to increased apoptosis through the accumulation of intracellular reactive oxygen species (ROS). Utsumi F *et al*. also reported that PAM treatment upregulated the fraction of intracellular ROS, resulting in apoptosis in ovarian cancer cells^10^. Taken together with our results, the induction of cell death by PAM treatment is a universal phenomenon in malignant cells.

In addition, we demonstrated that PAM induced autophagic cell death in endometrial cancer cells. PAM treatment increased the protein expression of LC3B in both HEC1A and HEC50 cells. Autophagy is a lysosome-mediated process that plays a crucial role in cellular homeostasis through the degradation of cytoplasmic components. Moreover, it is well known that autophagic cell death is one of the representative processes of cell death and is characterized by the absence of chromatin condensation and accompanied by autophagic vacuolization^27^. Hirst *et al*. previously reported that direct exposure to low temperature effectively induces necrosis and autophagic cell death in prostate cells due to increased DNA damage. To our knowledge, with the exception of apoptosis, the mechanism by which PAM induces cytotoxicity is not well understood. However, this study is the first report that raises autophagy as a mechanism of cell death by PAM.

With respect to mTOR pathway, we demonstrated that PAM led to a considerable decrease in phosphorylation of mTOR and AKT after 2–6 h treatment in AMEC cells. In HEC50 cells, phosphorylation of mTOR and AKT slightly decreased 1–6 h after PAM administration. This result is consistent with previous reports that suppression of mTOR pathway activates autophagy^28,29^. Interestingly, increased expression of LC3B protein was observed rapidly 0.5 hour after PAM treatment (Fig. 3E,F). Furthermore, the antiproliferative activity was observed even after a very short period of treatment (Fig. 1B,C). In addition, autophagy inhibitor, MHY1485, effectively rescued the reduced cell viability by PAM in both AMEC and HEC50 cells, however, no objective differences were found in phosphorylation of mTOR and AKT. Although our results do not reveal all the underlying mechanisms, this suggests that autophagic cell death in the short time after administration of PAM may not depend on inhibition of the mTOR pathway.

To conclude, PAM appears to inhibit cell viability while inducing autophagic cell death in endometrial cancer cells, which may provide a novel treatment for endometrial cancer. Further studies are needed to explore whether PAM treatment decreases the recurrence rate of peritoneal metastasis in endometrial cancer using an *in vivo* model.

## Materials and Methods

### Cells

Four endometrial cancer cell lines, AMEC, HEC50, ISHIKAWA, and RL95 were obtained from the American Type Culture Collection (ATCC, Manassas, VA, USA) and were maintained in RPMI-1640 medium (no. R8758, Sigma-Aldrich, St. Louis, MO, USA) with 10% heat-inactivated fetal bovine serum (FBS: Thermo Fisher Scientific, Yokohama, Japan) and 1% penicillin-streptomycin (Nacalai Tesque, Kyoto, Japan). All cells were cultured at 37 °C in a 5% CO_2_ humidified incubator.

### Materials

The structure of synthesized MHY1485 was obtained from Sigma (CAS 326914-06-1). Antibodies against LC3A/B (Cat. 4445, CST, Tokyo, Japan), mTOR (Cat. 2972, CST, Tokyo, Japan), phospho-mTOR (Ser2448; Cat. 39182, CST, Tokyo, Japan), Akt (Cat. 9272, CST, Tokyo, Japan), p-Akt (Ser473; Cat. 9271, CST, Tokyo, Japan), and p62/SQSTM1 (Cat. PM045, MBL, USA) were obtained, along with HRP-conjugated secondary antibodies from Cell Signal Technologies (CST, Tokyo, Japan).

### Experimental plasma system and PAM preparation

We utilized the NEAPP system as a plasma source with ultrahigh electron density (approximately 2 × 10^16^cm^−3^) (Fuji Corporation, Aichi, Japan)^30,31^. The operating conditions were as follows: an argon (Ar) gas flow mixed with oxygen and nitrogen gases (2 standard litres/min; slm), 15 kV of a 60-Hz ac power supply, 15 mm of the electrode distance, 20 mm of the slit length and a purge with an Ar gas (10 slm). We exposed the above NEAPP to 10 mL of RPMI-1640 medium without FBS in a 60-mm cell culture dish (AGC TECHNO GLASS CO., LTD, Shizuoka, Japan), which had been named PAM. The exposure distance between the exit of the plasma head and the medium surface (L) was 4 mm for all experiments.

### Cell viability assay

Cell lines (1 × 10^4^ cells) were first seeded into 96-well plates. After a 24-hour incubation period, the cells were treated with the appropriately diluted PAM for 24 h. Afterward, cell viability was assayed using the Cell Counting Kit-8 (DojinDo Molecular Technologies Inc, Maryland, USA), according to the manufacturer’s instructions. Absorbance was then measured at 450 nm using a microplate reader (Multiskan FC, Thermo Fisher Scientific K.K., Tokyo, Japan).

### Detection of intracellular ROS

Cells were seeded in a 24-well plate. On the following day, cells were washed once with PBS, and then 5-6-chloromethyl-2′7′-dichlorodihydroflorescein diacetate, acetyl ester (CM-H_2_DCFDA; Thermo Fisher Scientific, Yokohama, Japan) (4 mM) in PBS was loaded into cells for 15 minutes at 37 °C in the dark. CM-H_2_DCFDA was replaced with the appropriately diluted PAM or culture medium, and cells were incubated for 30 minutes at 37 °C, and observed using a BZ9000 microscope (Keyence, Osaka, Japan).

### Cell cycle analysis

Cells were seeded in a 12-well plate. On the following day, cells were treated with an appropriate concentration of PAM. After 24-hour, cells were harvested and fixed with ice-cold 70% ethanol for 1 hour at 4 °C. The cells were then washed twice with PBS and resuspended in a PI/RNase staining buffer (BD Pharmingen, NJ, USA) and incubated at RT for 15 min in the dark. The cells were analyzed by flow cytometry (BD FACSCanto II, NJ, USA) determine the cell cycle distribution and analyzed by ModFit LT™(Verity Software House, Inc., ME, USA).

### Western blotting

After treatment with the diluted PAM at each time course, the cells were washed with cold PBS and harvested using 5 x RIPA buffer [1 M Tris-HCl (pH 7.4), 5 M NaCl, 10% sodium deoxycholic acid, 125 mM EDTA] and protease inhibitor cocktail tablets (Roche Diagnostics, Indianapolis, IN, USA) on ice. Proteins were separated by SDS-PAGE gels, and then transferred onto Polyvinylidene difluoride (PVDF) membranes (Merk-Millipore, Tokyo, Japan). Membranes were incubated with various antibodies, then treated with HRP-conjugated secondary antibodies (GE Healthcare Life Sciences, Tokyo, Japan) at room temperature (RT) for 1 h. Bound antibodies were visualized using ECL^TM^ western blotting detection reagents (GE Healthcare, Backinghamshire, UK) and analyzed with an Image Quant LAS4000 Mini imaging system (GE). The antibodies used for immunoblotting were from the Autophagy Antibody Sampler Kit (1:1000; Cat. 4445, CST, Tokyo, Japan) and anti-GAPDH (1:1000; Cat.2118, CST Tokyo, Japan).

### Annexin V/7-AAD staining assay

Cells were cultured in 6-cm dish plates for 24 h before treatment with an appropriate concentration of PAM (1:8) for several incubation times. Cells were then trypsinized, washed twice with cold PBS, and then resuspended in 1X Binding buffer (BD Pharmingen, Cat. 51-66121E) at a concentration of 1 × 10^6^ cells/mL. Cells were then stained with APC-Annexin V (Cat. 550474, BD Pharmingen) and 7-AAD (Cat. 559925, BD Pharmingen), gently mixed and incubated at RT for 15 min in the dark. After the addition of 1X Binding buffer to each tube, the cells were analyzed by flow cytometry to determine the early/late apoptotic cell population. Cells cultured with Staurosporine (1 µM, 197-10251, Wako) at 37 °C for 6 h were used as the positive control.

### Immunofluorescence

Cells were cultured on glass coverslips in 6-well plates for 24 h before treatment with an appropriate concentration of PAM (1:4) for 1 h of incubation. Cells were then fixed with 100% methanol for 15 min at −20 °C, washed with PBS, and treated with blocking buffer [10% normal goat serum (Dako; Agilent Technologies, Inc., Santa Clara, CA, USA) and 0.3% Triton-X in PBS] for 1 h at RT and incubated overnight with the anti-LC3B (1:200) (Cat. 3868, Tokyo, Japan CST) antibody in blocking buffer at 4 °C. Cells were then washed three times with PBS and incubated with Alexa Fluor 594-conjugated secondary antibody (1:500) (Jackson ImmunoResearch, West Grove, PA, USA) for 1 h at RT and DAPI (1:500) in PBS for 5 min at RT. Finally, cells were observed using a fluorescence microscope (BX60; Olympus, Tokyo, JAPAN).

### Statistical analysis

All data are expressed as the mean ± SD. Statistical differences were analyzed using the Student’s *t*- test. *P* values of < 0.05 were considered statistically significant.

## Supplementary information

Supplementary Information.
