# Supplementary material for: Plasma-activated medium promotes autophagic cell death along with alteration of the mTOR pathway
Source: Sci Rep. 2020 Jan 31;10:1614. doi: 10.1038/s41598-020-58667-3 (PMC6994502; doi:10.1038/s41598-020-58667-3)
Supplement: Supplementary file 1 — Supplementary Information. [file 41598_2020_58667_MOESM1_ESM.docx]

Supplementary Information

**Plasma-activated medium promotes autophagic cell death**

**along with alteration of the mTOR pathway**

Nobuhisa Yoshikawa *^a^*, Wenting Liu *^b^*, Kae Nakamura *^a^*, Kosuke Yoshida *^a^*, Yoshiki Ikeda *^a^*, Hiromasa Tanaka *^c,d^*, Masaaki Mizuno *^d^*, Shinya Toyokuni *^e^*, Masaru Hori *^c^*, Fumitaka Kikkawa *^a^*, Hiroaki Kajiyama *^a^*

Corresponding author: Nobuhisa YOSHIKAWA (n-yoshikawa@med.nagoya-u.ac.jp)

*Department of Obstetrics and Gynecology, Nagoya University Graduate School of Medicine ^a^*

*Bell Research Center for Reproductive Health and Cancer, Nagoya University Graduate School of Medicine ^b^*

*Institute of Innovation for Future Society, Nagoya University ^c^*

*Center for Advanced Medicine and Clinical Research, Nagoya University Hospital ^d^*

*Department of Pathology and Biological Responses, Nagoya University Graduate School of Medicine ^e^*

**Supplementary Figure 1: Induction of G2/M-phase arrest in cancer cells by PAM.**

Cell cycle distribution of AMEC(A) and HEC50(B) cells were examined 24 h after PAM treatment by flow cytometry in different PAM concentrations. Statistical significances were observed the G2/M-phase arrest in all the PAM concentrations. NT, no treatment. Control, treatment with medium without plasma irradiation.


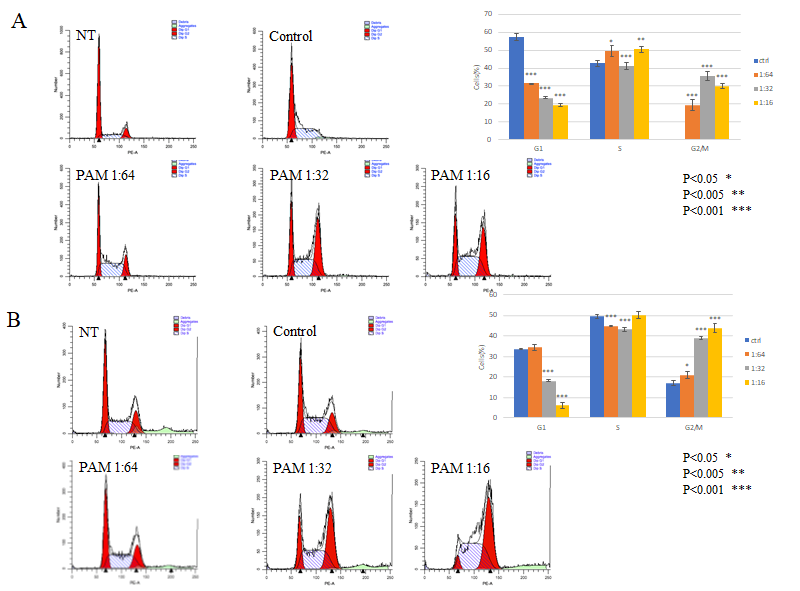


**Supplementary Figure 2: Intracellular ROS generation in AMEC cells teared with PAM.**

Cells were preloaded with CM-H2DCFDA for 15 min and then treated with different PAM concentrations. Images were visualized using a fluorescence microscope. Scale bar represents 100 μm.


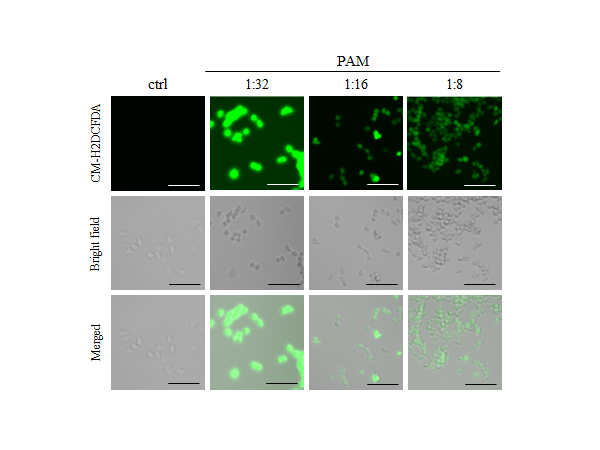


**Supplementary Figure 3: The original blots in the manuscript are presented.**

Figure 3B

Figure 3C

Figure 3D

Figure 3E

Figure 3F

Figure 4C

Figure 4D
